# Diaqua­{6,6′-dimeth­oxy-2,2′-[ethane-1,2-diylbis(nitrilo­methyl­idyne)]diphenolato-κ^2^ *O*,*N*,*N*′,*O*′}manganese(III) perchlorate 18-crown-6 hemisolvate monohydrate

**DOI:** 10.1107/S1600536808044176

**Published:** 2009-01-08

**Authors:** Zhan-Xian Li, Xia Li, Li-Feng Zhang, Ming-Ming Yu

**Affiliations:** a Department of Chemistry, Zhengzhou University, Zhengzhou 450001, People’s Republic of China; b College of Polymer Science and Engineering, Sichuan University, Chengdu 610065, People’s Republic of China

## Abstract

In the cation of the title compound, [Mn(C_18_H_18_N_2_O_4_)(H_2_O)_2_]ClO_4_·0.5C_12_H_24_O_6_·H_2_O, the Mn^III^ ion is coordinated by two water O atoms, and two O atoms and two N atoms from the tetradentate 6,6′-dimeth­oxy-2,2′-[ethane-1,2-diylbis(nitrilo­methyl­idyne)]di­phenolate ligand, completing a distorted octa­hedral geometry. One O atom of the 18-crown-6-ether is disordered over two positions with occupancies of 0.70 (2) and 0.30 (2).

## Related literature

For background on manganese-containing complexes, see: Garnovskii *et al.* (1993 ▶); Huang *et al.* (2002 ▶); For related structures, see: Christou (1989 ▶); Yu *et al.* (2007 ▶). 
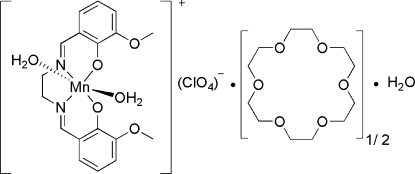


## Experimental

### 

#### Crystal data

[Mn(C_18_H_18_N_2_O_4_)(H_2_O)_2_]ClO_4_·0.5C_12_H_24_O_6_·H_2_O
*M* *_r_* = 666.94
Monoclinic, 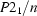

*a* = 11.7287 (12) Å
*b* = 15.5814 (16) Å
*c* = 16.8158 (16) Å
β = 105.529 (2)°
*V* = 2960.9 (5) Å^3^
*Z* = 4
Mo *K*α radiation
μ = 0.61 mm^−1^
*T* = 272 (3) K
0.40 × 0.35 × 0.25 mm

#### Data collection

Bruker SMART 1K CCD area-detector diffractometer
Absorption correction: multi-scan (*SADABS*; Bruker, 2000 ▶) *T*_min_ = 0.781, *T*_max_ = 0.857
14526 measured reflections
5198 independent reflections
3798 reflections with *I* > 2σ(*I*)
*R*_int_ = 0.034

#### Refinement

*R*[*F* ^2^ > 2σ(*F* ^2^)] = 0.077
*wR*(*F* ^2^) = 0.239
*S* = 1.16
5198 reflections
382 parameters
1 restraint
H-atom parameters constrained
Δρ_max_ = 1.71 e Å^−3^
Δρ_min_ = −0.99 e Å^−3^

### 

Data collection: *SMART* (Bruker, 2000 ▶); cell refinement: *SAINT* (Bruker, 2000 ▶); data reduction: *SAINT*; program(s) used to solve structure: *SHELXTL* (Sheldrick, 2008 ▶); program(s) used to refine structure: *SHELXTL*; molecular graphics: *SHELXTL*; software used to prepare material for publication: *SHELXTL*.

## Supplementary Material

Crystal structure: contains datablocks I, global. DOI: 10.1107/S1600536808044176/hg2451sup1.cif

Structure factors: contains datablocks I. DOI: 10.1107/S1600536808044176/hg2451Isup2.hkl

Additional supplementary materials:  crystallographic information; 3D view; checkCIF report

## Figures and Tables

**Fig. 1.**
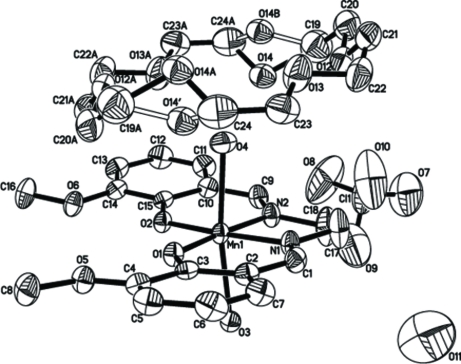
A view of complex (I), showing 30% probability displacement ellipsoids and the atom-numbering scheme. H atoms have been omitted for clarity.

## supplementary crystallographic information

### Comment

Manganese can exhibit several oxidation states and may provide the basis of models for active sites of biological systems such as the oxygen-evolving complex of photosystem and enzymes like superoxide dismutase, catalase, arginase (Garnovskii, *et al.*, 1993; Huang, *et al.*, 2002). The progress in elucidating the strutural and functional aspects of manganese-containing systems, has essentially been connected to the vast number of inorganic model complexes reported during the last few decades (Christou, 1989; Yu, *et al.*, 2007). Here, the synthesis and crystal structure of the title complex (I) are reported.

The title complex (I) comprises one [Mn(C_18_H_18_N_2_O_4_)(H_2_O)_2_]^+^ cation, one perchlorate anion, half 18-crown-6 and one uncoordinated water molecule. (Fig. 1). The Mn^III^ ion in each [Mn(C_18_H_18_N_2_O_4_)(H_2_O)_2_]^+^ cation is coordinated by two O atoms from water molecules, two O atoms and two N atoms from 6,6'-dimethoxy-2,2'-(ethane-1,2-diyldiiminodimethylene)diphenol ligands, completing a distorted octahedral geometry. Mn—N bond lengths are 1.973 (13), 1.969 (13) Å, respectly. The Mn—O bond distances to the 6,6'-dimethoxy-2,2'-(ethane-1,2-diyldiiminodimethylene)diphenol ligand are each 1.876 (10) Å. They are much shorter than the Mn—O bond distances of coordinated water (Mn1—O3 = 2.274 (11) Å, Mn1—O4 = 2.270 (12)Å).

### Experimental

A mixture of manganese(II) perchlorate (1 mmol, 253.8 mg), 6,6'-dimethoxy-2,2'-(ethane-1,2-diyldiiminodimethylene)diphenol (1 mmol, 326.4 mg) in 40 ml me thanol and 18-crown-6-ether (2 mmol, 528.6 mg) in water 40 ml was refluxed for one hour. The solution was cooled and filtrated. Crystals suitable for X-ray diffraction analysis were obtained by slow evaparation at room temperature for three weeks.

### Refinement

All H atoms were placed in geometrically calculated positions with C—H = 0.97 Å for CH_2_ H atoms, C—H = 0.93 Å for aromatic and CH H atoms and 0.82 Å for N—H H atoms and were refined isotropic with *U*_iso_(H) = 1.2*U*_eq_(C) of parent atom using a riding model. H atoms of H_2_O were located from difference maps and refined with a distance restraint O—H = 0.82 (1) Å. *U*_iso_(H) = 1.2*U*_eq_(O). *DFIX* restraints were applied to O···H distances of [1.97 (1) Å] H24*a* and O14 to remain 18-crown-6 molecule in normal format.

### Crystal data

**Table:**

[Mn(C_18_H_18_N_2_O_4_)(H_2_O)_2_]ClO_4_·0.5C_12_H_24_O_6_·H_2_O	*F*(000) = 1392
*M**_r_* = 666.94	*D*_x_ = 1.496 Mg m^−^^3^
Monoclinic, *P*2_1_/*n*	Mo *K*α radiation, λ = 0.71073 Å
Hall symbol: -P 2yn	Cell parameters from 5198 reflections
*a* = 11.7287 (12) Å	θ = 1.8–25.0°
*b* = 15.5814 (16) Å	µ = 0.61 mm^−^^1^
*c* = 16.8158 (16) Å	*T* = 272 K
β = 105.529 (2)°	Block, brown
*V* = 2960.9 (5) Å^3^	0.40 × 0.35 × 0.25 mm
*Z* = 4	

### Data collection

**Table:**

Bruker SMART 1K CCD area-detector diffractometer	5198 independent reflections
Radiation source: fine-focus sealed tube	3798 reflections with *I* > 2σ(*I*)
graphite	*R*_int_ = 0.034
φ and ω scans	θ_max_ = 25.0°, θ_min_ = 1.8°
Absorption correction: multi-scan (*SADABS*; Bruker, 2000)	*h* = −13→13
*T*_min_ = 0.781, *T*_max_ = 0.857	*k* = −18→18
14526 measured reflections	*l* = −16→20

### Refinement

**Table:**

Refinement on *F*^2^	Primary atom site location: structure-invariant direct methods
Least-squares matrix: full	Secondary atom site location: difference Fourier map
*R*[*F*^2^ > 2σ(*F*^2^)] = 0.077	Hydrogen site location: inferred from neighbouring sites
*wR*(*F*^2^) = 0.239	H-atom parameters constrained
*S* = 1.16	*w* = 1/[σ^2^(*F*_o_^2^) + (0.1233*P*)^2^ + 3.531*P*] where *P* = (*F*_o_^2^ + 2*F*_c_^2^)/3
5198 reflections	(Δ/σ)_max_ = 0.039
382 parameters	Δρ_max_ = 1.71 e Å^−^^3^
1 restraint	Δρ_min_ = −0.99 e Å^−^^3^

### Special details

**Table:**

Geometry. All e.s.d.'s (except the e.s.d. in the dihedral angle between two l.s. planes) are estimated using the full covariance matrix. The cell e.s.d.'s are taken into account individually in the estimation of e.s.d.'s in distances, angles and torsion angles; correlations between e.s.d.'s in cell parameters are only used when they are defined by crystal symmetry. An approximate (isotropic) treatment of cell e.s.d.'s is used for estimating e.s.d.'s involving l.s. planes.
Refinement. Refinement of *F*^2^ against ALL reflections. The weighted *R*-factor *wR* and goodness of fit *S* are based on *F*^2^, conventional *R*-factors *R* are based on *F*, with *F* set to zero for negative *F*^2^. The threshold expression of *F*^2^ > σ(*F*^2^) is used only for calculating *R*-factors(gt) *etc*. and is not relevant to the choice of reflections for refinement. *R*-factors based on *F*^2^ are statistically about twice as large as those based on *F*, and *R*- factors based on ALL data will be even larger.

### Fractional atomic coordinates and isotropic or equivalent isotropic displacement parameters (Å^2^)

**Table:**

	*x*	*y*	*z*	*U*_iso_*/*U*_eq_	Occ. (<1)
Mn1	0.8434 (2)	0.55184 (14)	0.55021 (12)	0.0362 (9)	
Cl1	0.7718 (11)	0.9782 (5)	0.5697 (5)	0.121 (3)	
O1	0.8797 (10)	0.4346 (6)	0.5501 (6)	0.041 (2)	
O2	0.8398 (9)	0.5661 (6)	0.4388 (6)	0.040 (2)	
O3	1.0384 (9)	0.5863 (7)	0.5967 (6)	0.044 (3)	
H1O3	1.0942	0.5502	0.6017	0.053*	
H2O3	1.0556	0.6301	0.5721	0.053*	
O4	0.6460 (10)	0.5261 (9)	0.5206 (8)	0.061 (3)	
H204	0.6098	0.5451	0.4755	0.073*	
H104	0.6525	0.4730	0.5233	0.073*	
O5	0.9243 (10)	0.2794 (7)	0.5141 (7)	0.050 (3)	
O6	0.8251 (11)	0.5434 (7)	0.2837 (6)	0.053 (3)	
O7	0.767 (3)	1.0480 (16)	0.616 (2)	0.194 (13)	
O8	0.747 (4)	0.978 (3)	0.4889 (16)	0.235 (17)	
O9	0.900 (5)	0.962 (4)	0.588 (3)	0.29 (2)	
O10	0.723 (4)	0.9145 (17)	0.598 (2)	0.225 (16)	
O11	0.996 (4)	0.582 (3)	0.901 (3)	0.28 (2)	
H1O1	0.9215	0.5744	0.8843	0.339*	
H2O1	1.0292	0.5338	0.9001	0.339*	
O12	0.4518 (12)	0.6187 (9)	0.6164 (8)	0.070 (4)	
O13	0.4881 (17)	0.4407 (11)	0.6454 (10)	0.094 (5)	
O14	0.501 (4)	0.667 (3)	0.468 (3)	0.081 (5)	0.30 (2)
O14'	0.5994 (19)	0.3432 (15)	0.5508 (14)	0.081 (5)	0.70 (2)
N1	0.8421 (13)	0.5487 (8)	0.6673 (8)	0.046 (3)	
N2	0.8077 (13)	0.6740 (8)	0.5613 (8)	0.048 (3)	
C1	0.8610 (15)	0.4826 (11)	0.7146 (10)	0.049 (4)	
H1	0.8574	0.4901	0.7687	0.059*	
C2	0.8873 (14)	0.3984 (10)	0.6905 (9)	0.043 (4)	
C3	0.8943 (12)	0.3779 (9)	0.6106 (9)	0.038 (3)	
C4	0.9174 (13)	0.2922 (10)	0.5928 (9)	0.042 (4)	
C5	0.9298 (16)	0.2298 (11)	0.6522 (11)	0.054 (4)	
H5	0.9434	0.1733	0.6392	0.065*	
C6	0.9224 (17)	0.2496 (12)	0.7313 (12)	0.061 (5)	
H6	0.9314	0.2066	0.7709	0.074*	
C7	0.9019 (16)	0.3324 (12)	0.7510 (11)	0.055 (4)	
H7	0.8974	0.3455	0.8040	0.066*	
C8	0.940 (2)	0.1927 (11)	0.4890 (12)	0.067 (5)	
H8A	1.0114	0.1694	0.5239	0.100*	
H8B	0.9438	0.1926	0.4328	0.100*	
H8C	0.8737	0.1584	0.4937	0.100*	
C9	0.7780 (16)	0.7286 (10)	0.5019 (10)	0.049 (4)	
H9	0.7603	0.7839	0.5156	0.059*	
C10	0.7695 (15)	0.7122 (10)	0.4161 (10)	0.046 (4)	
C11	0.7284 (17)	0.7785 (11)	0.3594 (11)	0.057 (5)	
H11	0.7083	0.8312	0.3778	0.068*	
C12	0.7179 (18)	0.7665 (12)	0.2782 (12)	0.065 (5)	
H12	0.6893	0.8105	0.2409	0.077*	
C13	0.7498 (16)	0.6887 (12)	0.2505 (10)	0.056 (5)	
H13	0.7435	0.6814	0.1946	0.067*	
C14	0.7909 (14)	0.6217 (10)	0.3045 (9)	0.044 (4)	
C15	0.8008 (13)	0.6326 (9)	0.3896 (9)	0.037 (3)	
C16	0.815 (2)	0.5265 (14)	0.1996 (11)	0.075 (6)	
H16A	0.7329	0.5305	0.1690	0.112*	
H16B	0.8434	0.4699	0.1938	0.112*	
H16C	0.8601	0.5678	0.1787	0.112*	
C17	0.810 (3)	0.6315 (14)	0.6966 (13)	0.090 (7)	
H17A	0.7287	0.6284	0.7004	0.108*	
H17B	0.8603	0.6422	0.7516	0.108*	
C18	0.820 (3)	0.6989 (15)	0.6465 (13)	0.096 (8)	
H18A	0.8960	0.7260	0.6683	0.115*	
H18B	0.7591	0.7410	0.6477	0.115*	
C19	0.424 (3)	0.726 (2)	0.516 (2)	0.117 (8)	
H19A	0.4749	0.7722	0.5444	0.140*	
H19B	0.3598	0.7528	0.4747	0.140*	
C20	0.375 (3)	0.6846 (18)	0.5747 (17)	0.101 (7)	
H20A	0.2984	0.6600	0.5466	0.121*	
H20B	0.3621	0.7262	0.6145	0.121*	
C21	0.402 (2)	0.5692 (18)	0.6685 (16)	0.089 (7)	
H21A	0.3270	0.5452	0.6368	0.107*	
H21B	0.3860	0.6055	0.7113	0.107*	
C22	0.481 (2)	0.5008 (17)	0.7058 (14)	0.087 (7)	
H22A	0.5585	0.5240	0.7315	0.105*	
H22B	0.4516	0.4731	0.7482	0.105*	
C23	0.5581 (16)	0.3749 (14)	0.6760 (12)	0.083 (7)	
H23A	0.5261	0.3457	0.7162	0.100*	
H23B	0.6354	0.3970	0.7050	0.100*	
C24	0.5727 (16)	0.3147 (14)	0.6174 (12)	0.104 (9)	
H24A	0.5004	0.2814	0.5993	0.124*	
H24B	0.6356	0.2754	0.6441	0.124*	

### Atomic displacement parameters (Å^2^)

**Table:**

	*U*^11^	*U*^22^	*U*^33^	*U*^12^	*U*^13^	*U*^23^
Mn1	0.0466 (15)	0.0324 (13)	0.0297 (13)	0.0031 (9)	0.0103 (10)	0.0027 (9)
Cl1	0.209 (10)	0.083 (5)	0.078 (5)	−0.017 (5)	0.051 (5)	0.001 (4)
O1	0.055 (6)	0.036 (5)	0.034 (5)	0.002 (5)	0.013 (5)	0.007 (4)
O2	0.053 (6)	0.037 (5)	0.029 (5)	0.006 (5)	0.008 (4)	0.005 (4)
O3	0.047 (6)	0.042 (6)	0.043 (6)	0.001 (5)	0.011 (5)	0.001 (5)
O4	0.047 (7)	0.075 (8)	0.058 (8)	−0.004 (6)	0.009 (6)	0.006 (6)
O5	0.066 (7)	0.033 (6)	0.049 (7)	0.001 (5)	0.015 (5)	0.002 (5)
O6	0.075 (8)	0.050 (7)	0.033 (6)	−0.005 (6)	0.014 (5)	0.001 (5)
O7	0.27 (4)	0.104 (18)	0.22 (3)	−0.020 (19)	0.08 (3)	−0.052 (18)
O8	0.33 (5)	0.28 (4)	0.079 (16)	−0.09 (3)	0.03 (2)	0.03 (2)
O9	0.28 (4)	0.36 (5)	0.23 (4)	0.08 (4)	0.08 (4)	−0.07 (3)
O10	0.43 (5)	0.095 (16)	0.22 (3)	−0.03 (2)	0.20 (3)	−0.001 (18)
O11	0.27 (5)	0.25 (5)	0.30 (5)	−0.01 (4)	0.04 (4)	0.01 (4)
O12	0.068 (9)	0.077 (9)	0.060 (8)	0.004 (7)	0.008 (7)	−0.020 (7)
O13	0.121 (14)	0.089 (12)	0.064 (10)	0.015 (10)	0.010 (9)	−0.001 (8)
O14	0.063 (10)	0.088 (12)	0.091 (12)	−0.001 (10)	0.021 (10)	−0.005 (10)
O14'	0.063 (10)	0.088 (12)	0.091 (12)	−0.001 (10)	0.021 (10)	−0.005 (10)
N1	0.065 (9)	0.043 (7)	0.032 (7)	0.005 (6)	0.017 (6)	0.001 (6)
N2	0.069 (9)	0.036 (7)	0.040 (7)	0.010 (6)	0.017 (6)	0.001 (6)
C1	0.059 (10)	0.056 (10)	0.036 (8)	0.002 (8)	0.018 (7)	0.006 (8)
C2	0.044 (9)	0.044 (9)	0.040 (8)	0.002 (7)	0.010 (7)	0.011 (7)
C3	0.031 (7)	0.040 (8)	0.041 (8)	−0.003 (6)	0.005 (6)	0.007 (6)
C4	0.040 (8)	0.040 (8)	0.043 (9)	−0.003 (7)	0.008 (7)	0.006 (7)
C5	0.062 (11)	0.038 (9)	0.062 (11)	0.007 (8)	0.014 (9)	0.017 (8)
C6	0.068 (12)	0.058 (11)	0.060 (11)	0.008 (9)	0.020 (9)	0.029 (9)
C7	0.061 (11)	0.059 (11)	0.047 (10)	0.004 (9)	0.017 (8)	0.022 (8)
C8	0.088 (15)	0.037 (9)	0.072 (13)	−0.003 (9)	0.017 (11)	−0.007 (9)
C9	0.065 (11)	0.032 (8)	0.053 (10)	0.009 (7)	0.021 (8)	0.001 (7)
C10	0.053 (10)	0.038 (8)	0.046 (9)	−0.001 (7)	0.012 (7)	0.008 (7)
C11	0.068 (12)	0.042 (9)	0.061 (11)	0.011 (8)	0.017 (9)	0.018 (8)
C12	0.077 (13)	0.055 (11)	0.058 (12)	0.011 (10)	0.011 (10)	0.028 (9)
C13	0.066 (11)	0.060 (11)	0.038 (9)	−0.003 (9)	0.009 (8)	0.020 (8)
C14	0.047 (9)	0.043 (9)	0.039 (8)	−0.007 (7)	0.008 (7)	0.009 (7)
C15	0.036 (8)	0.036 (8)	0.037 (8)	−0.003 (6)	0.006 (6)	0.007 (6)
C16	0.113 (18)	0.071 (13)	0.040 (10)	−0.010 (12)	0.019 (11)	−0.009 (9)
C17	0.16 (2)	0.062 (12)	0.057 (11)	0.031 (13)	0.049 (13)	0.002 (10)
C18	0.17 (2)	0.064 (12)	0.061 (12)	0.030 (14)	0.039 (14)	0.000 (10)
C19	0.118 (8)	0.116 (9)	0.116 (8)	0.003 (3)	0.030 (4)	−0.001 (3)
C20	0.095 (13)	0.097 (14)	0.103 (14)	0.022 (12)	0.014 (11)	−0.034 (12)
C21	0.080 (14)	0.099 (16)	0.091 (16)	−0.029 (13)	0.027 (13)	−0.041 (14)
C22	0.107 (19)	0.101 (19)	0.059 (13)	−0.034 (16)	0.030 (13)	−0.014 (13)
C23	0.074 (15)	0.12 (2)	0.061 (13)	0.011 (14)	0.020 (11)	0.020 (14)
C24	0.093 (19)	0.093 (19)	0.12 (2)	0.022 (15)	0.024 (16)	0.043 (17)

### Geometric parameters (Å, °)

**Table:**

Mn1—O2	1.876 (10)	C6—C7	1.37 (3)
Mn1—O1	1.876 (10)	C6—H6	0.9300
Mn1—N2	1.969 (13)	C7—H7	0.9300
Mn1—N1	1.973 (12)	C8—H8A	0.9600
Mn1—O4	2.270 (12)	C8—H8B	0.9600
Mn1—O3	2.274 (11)	C8—H8C	0.9600
Cl1—O10	1.30 (3)	C9—C10	1.44 (2)
Cl1—O8	1.31 (3)	C9—H9	0.9300
Cl1—O7	1.34 (3)	C10—C15	1.40 (2)
Cl1—O9	1.47 (5)	C10—C11	1.40 (2)
O1—C3	1.323 (17)	C11—C12	1.35 (3)
O2—C15	1.328 (17)	C11—H11	0.9300
O3—H1O3	0.8500	C12—C13	1.39 (3)
O3—H2O3	0.8500	C12—H12	0.9300
O4—H204	0.8189	C13—C14	1.38 (2)
O4—H104	0.8304	C13—H13	0.9300
O5—C4	1.362 (18)	C14—C15	1.41 (2)
O5—C8	1.44 (2)	C16—H16A	0.9600
O6—C14	1.359 (19)	C16—H16B	0.9600
O6—C16	1.41 (2)	C16—H16C	0.9600
O11—H1O1	0.8505	C17—C18	1.37 (3)
O11—H2O1	0.8498	C17—H17A	0.9700
O12—C21	1.41 (3)	C17—H17B	0.9700
O12—C20	1.42 (3)	C18—H18A	0.9700
O13—C23	1.33 (2)	C18—H18B	0.9700
O13—C22	1.40 (3)	C19—C20	1.43 (4)
O14—C24^i^	1.50 (5)	C19—O14'^i^	1.53 (4)
O14—C19	1.65 (6)	C19—H19A	0.9700
O14'—C24	1.32 (3)	C19—H19B	0.9700
O14'—C19^i^	1.53 (4)	C20—H20A	0.9700
N1—C1	1.28 (2)	C20—H20B	0.9700
N1—C17	1.47 (2)	C21—C22	1.44 (4)
N2—C9	1.29 (2)	C21—H21A	0.9700
N2—C18	1.46 (2)	C21—H21B	0.9700
C1—C2	1.43 (2)	C22—H22A	0.9700
C1—H1	0.9300	C22—H22B	0.9700
C2—C3	1.41 (2)	C23—C24	1.4037
C2—C7	1.42 (2)	C23—H23A	0.9700
C3—C4	1.41 (2)	C23—H23B	0.9700
C4—C5	1.37 (2)	C24—O14^i^	1.50 (5)
C5—C6	1.39 (3)	C24—H24A	0.9700
C5—H5	0.9300	C24—H24B	0.9700
			
O2—Mn1—O1	93.4 (4)	C12—C11—H11	119.8
O2—Mn1—N2	91.8 (5)	C10—C11—H11	119.7
O1—Mn1—N2	174.8 (5)	C11—C12—C13	120.1 (16)
O2—Mn1—N1	174.4 (5)	C11—C12—H12	119.9
O1—Mn1—N1	92.2 (5)	C13—C12—H12	119.9
N2—Mn1—N1	82.6 (5)	C14—C13—C12	121.2 (16)
O2—Mn1—O4	93.0 (5)	C14—C13—H13	119.4
O1—Mn1—O4	92.6 (5)	C12—C13—H13	119.4
N2—Mn1—O4	87.8 (6)	O6—C14—C13	125.7 (15)
N1—Mn1—O4	86.2 (5)	O6—C14—C15	114.7 (13)
O2—Mn1—O3	93.8 (4)	C13—C14—C15	119.6 (16)
O1—Mn1—O3	91.3 (4)	O2—C15—C10	124.6 (13)
N2—Mn1—O3	87.6 (5)	O2—C15—C14	117.3 (13)
N1—Mn1—O3	86.5 (5)	C10—C15—C14	118.1 (13)
O4—Mn1—O3	171.9 (4)	O6—C16—H16A	109.4
O10—Cl1—O8	112 (2)	O6—C16—H16B	109.5
O10—Cl1—O7	108 (2)	H16A—C16—H16B	109.5
O8—Cl1—O7	125 (3)	O6—C16—H16C	109.5
O10—Cl1—O9	109 (3)	H16A—C16—H16C	109.5
O8—Cl1—O9	98 (3)	H16B—C16—H16C	109.5
O7—Cl1—O9	102 (3)	C18—C17—N1	113.2 (17)
C3—O1—Mn1	129.2 (10)	C18—C17—H17A	108.9
C15—O2—Mn1	128.8 (9)	N1—C17—H17A	108.9
Mn1—O3—H1O3	123.7	C18—C17—H17B	108.9
Mn1—O3—H2O3	111.7	N1—C17—H17B	108.9
H1O3—O3—H2O3	107.7	H17A—C17—H17B	107.8
Mn1—O4—H204	112.6	C17—C18—N2	113.6 (19)
Mn1—O4—H104	95.2	C17—C18—H18A	108.8
H204—O4—H104	115.4	N2—C18—H18A	108.9
C4—O5—C8	117.8 (13)	C17—C18—H18B	108.8
C14—O6—C16	118.2 (14)	N2—C18—H18B	108.8
H1O1—O11—H2O1	107.8	H18A—C18—H18B	107.7
C21—O12—C20	113 (2)	C20—C19—O14'^i^	99 (3)
C23—O13—C22	113 (2)	C20—C19—O14	117 (3)
C24^i^—O14—C19	96 (3)	C20—C19—H19A	107.2
C24—O14'—C19^i^	110 (2)	O14'^i^—C19—H19A	148.9
C1—N1—C17	120.9 (14)	O14—C19—H19A	108.0
C1—N1—Mn1	126.3 (11)	C20—C19—H19B	108.0
C17—N1—Mn1	112.7 (11)	O14'^i^—C19—H19B	80.4
C9—N2—C18	121.6 (14)	O14—C19—H19B	108.0
C9—N2—Mn1	125.9 (11)	H19A—C19—H19B	106.8
C18—N2—Mn1	112.5 (11)	C19—C20—O12	110 (2)
N1—C1—C2	125.1 (14)	C19—C20—H20A	109.7
N1—C1—H1	117.5	O12—C20—H20A	109.7
C2—C1—H1	117.4	C19—C20—H20B	109.5
C3—C2—C1	123.5 (13)	O12—C20—H20B	109.6
C3—C2—C7	119.4 (15)	H20A—C20—H20B	108.1
C1—C2—C7	117.1 (15)	O12—C21—C22	110 (2)
O1—C3—C2	123.7 (14)	O12—C21—H21A	109.6
O1—C3—C4	117.5 (13)	C22—C21—H21A	109.6
C2—C3—C4	118.8 (13)	O12—C21—H21B	109.5
O5—C4—C5	125.4 (15)	C22—C21—H21B	109.6
O5—C4—C3	114.2 (13)	H21A—C21—H21B	108.1
C5—C4—C3	120.4 (15)	O13—C22—C21	110 (2)
C4—C5—C6	121.1 (16)	O13—C22—H22A	109.7
C4—C5—H5	119.5	C21—C22—H22A	109.7
C6—C5—H5	119.4	O13—C22—H22B	109.8
C7—C6—C5	120.0 (16)	C21—C22—H22B	109.7
C7—C6—H6	120.0	H22A—C22—H22B	108.2
C5—C6—H6	120.0	O13—C23—C24	115.01
C6—C7—C2	120.3 (17)	O13—C23—H23A	108.4
C6—C7—H7	119.9	C24—C23—H23A	108.5
C2—C7—H7	119.9	O13—C23—H23B	108.7
O5—C8—H8A	109.5	C24—C23—H23B	108.5
O5—C8—H8B	109.5	H23A—C23—H23B	107.5
H8A—C8—H8B	109.5	O14^i^—C24—O14'	47 (2)
O5—C8—H8C	109.5	O14^i^—C24—C23	113 (2)
H8A—C8—H8C	109.5	O14'—C24—C23	118.2 (14)
H8B—C8—H8C	109.5	O14^i^—C24—H24A	64.5
N2—C9—C10	126.2 (14)	O14'—C24—H24A	107.3
N2—C9—H9	116.9	C23—C24—H24A	108.5
C10—C9—H9	116.9	O14^i^—C24—H24B	137.7
C15—C10—C11	120.5 (15)	O14'—C24—H24B	106.3
C15—C10—C9	121.5 (14)	C23—C24—H24B	108.5
C11—C10—C9	118.0 (15)	H24A—C24—H24B	107.5
C12—C11—C10	120.5 (17)		

Symmetry codes: (i) −*x*+1, −*y*+1, −*z*+1.
